# Novel estimation of tomato soluble solids content using linearly transformed reflectance-based spectral indices

**DOI:** 10.3389/fpls.2026.1729375

**Published:** 2026-02-05

**Authors:** Naisen Liu, Yuan Fang, Yongzhen Zhao, Yuezhen Chen, Yangxia Zheng, Xuedong Zha, Jingyu Guo, Xia Li

**Affiliations:** 1 School of Life Science, Huaiyin Normal University, Huai’an, China; 2 Huai’an Institute of Vegetable Sciences, Huai’an, China; 3 College of Horticulture, Sichuan Agricultural University, Chengdu, China; 4 Huai’an Agricultural Information Center, Huai’an, China

**Keywords:** genetic algorithm, hyperspectral spectroscopy, linearly transformed indices, spectral indices, spectral reflectance landscape, tomato SSC

## Abstract

Rapid and non-destructive estimation of soluble solids content (SSC) is essential for tomato quality evaluation, yet the generalization ability of many existing spectral models remains limited when applied across multiple cultivars. In this study, hyperspectral reflectance was combined with a genetic algorithm (GA)–based optimization strategy to develop a robust SSC prediction framework applicable to diverse tomato types. Spectral reflectance and SSC (°Brix) were measured for 152 fruits representing 13 cultivars, including large red, medium red, red cherry, and yellow cherry types. To overcome the structural rigidity of conventional fixed-form spectral indices, reflectance spectra were linearly transformed to construct three novel indices: the linearly transformed difference spectral index (ltDSI), linearly transformed normalized difference spectral index (ltNDSI), and linearly transformed ratio spectral index (ltRSI). For each index, GA was employed to simultaneously optimize wavelength combinations and transformation coefficients. Under identical calibration and validation datasets, the GA-optimized indices consistently outperformed conventional two-band spectral indices as well as full-spectrum partial least squares models, while exhibiting markedly reduced sensitivity to tomato type. Across all validation datasets, the proposed models achieved coefficients of determination of approximately 0.80, with root mean square errors around 0.6°Brix and mean relative errors close to 10%. These results demonstrate that joint optimization of spectral index structure and parameters is an effective strategy for improving model robustness and transferability. The proposed framework provides a scalable solution for non-destructive SSC assessment and offers practical guidance for the development of low-cost, field-deployable spectral sensing tools for fruit quality phenotyping across cultivars and growing conditions.

## 1 Introduction

Tomato (*Solanum lycopersicum* L.) is one of the most widely cultivated horticultural crops, is valued for its flavor, nutritional composition, and medicinal properties (Friedman, 2013, 2015; Kwon et al., 2024). Among its quality attributes, the soluble solids content (SSC) is a key indicator influencing consumer preference and marketability (Xia et al., 2020). The accurate assessment of SSC is therefore of great significance for crop management (Wang F. et al., 2022), postharvest grading (Tang et al., 2018), consumer choice, and food processing (Kim et al., 2023).

Conventional methods for measuring SSC, such as high- performance liquid chromatography (Ali et al., 2022) and refractometry (Jaywant et al., 2022), provide accurate results. However, their destructive sampling and low measurement efficiency limit their practical use. Spectroscopy has emerged as a powerful nondestructive technique for assessing SSC and other fruit quality attributes (Lu et al., 2000; Rongtong et al., 2018; Li S. et al., 2024). Despite its advantages, several challenges remain. Fruit spectra are strongly affected by fruit size and internal structure, and the generalization ability of existing models across cultivars is often limited. Fruit spectral measurements typically rely on reflectance (Lu, 2001; He et al., 2005) or transmittance (Khuriyati and Matsuoka, 2004; Tian et al., 2007). Transmittance spectroscopy enables deeper light–tissue interactions and can capture SSC information more effectively (Tian et al., 2023). However, its penetration is weak for large fruits (Xu et al., 2024), reducing measurement accuracy. Even in small fruits, variability in internal structure introduces substantial interference. Tomatoes contain complex internal partitions (Khuriyati and Matsuoka, 2004), grapes contain seeds, and peaches have large pits. These structural differences make the optimal measurement position difficult to define, especially because internal features are invisible. Transmittance measurements also require sealed, light-proof configurations (Cai et al., 2024; Zheng et al., 2024). Fruits must be placed in dedicated holders, which limits measurement speed and makes on-plant assessment nearly impossible. By contrast, reflectance spectroscopy captures only surface information and is unaffected by internal structure, but it contains less SSC-related information, imposing higher demands on model construction.

The adaptability of prediction models to different cultivars is another critical concern because it determines their practical utility. Many studies use only one cultivar (He et al., 2005; Radzevičius et al., 2016) or a few cultivars (Li S. et al., 2024), providing limited evidence of model generalization. Choi et al. (2017) reported cross-cultivar transferability in Asian pears, whereas studies on sugarcane (Chea et al., 2020), melon (Kusumiyati et al., 2022), and tomato (Ibáñez et al., 2019) demonstrated cultivar-specific prediction accuracy. These inconsistent findings may result from small sample diversity or differences in cultivar similarity. Evidence from homogenized tomato samples supports this view: models built using puree and juice spectra successfully predicted SSC across 53 genetically diverse cultivars (Sun et al., 2021). This highlights the need for prediction models developed using intact fruits from multiple types and diverse cultivars.

Current modeling approaches for fruit quality prediction rely on spectral indices or machine learning methods such as partial least squares regression (PLS) and random forests (Li H. et al., 2024; Elsayed et al., 2025). Although machine learning models often achieve high accuracy, they typically require computationally intensive processing or numerous spectral bands, which increases hardware cost and limits sensor integration. Spectral-index-based models use fewer bands and are computationally simple, making them more suitable for embedded sensors. However, their accuracy can be limited and must be improved. Conventional two-band indices, such as the difference spectral index (DSI), normalized difference spectral index (NDSI), and ratio spectral index (RSI), remain widely used, but recent evidence indicates that newly designed indices can further enhance SSC prediction (Wang Q. et al., 2022). Selecting sensitive wavelengths from a large spectral range remains challenging because of the vast number of possible band combinations. Genetic algorithms (GA), which simulate evolutionary processes such as selection, crossover, and mutation, provide an effective global optimization strategy (Shahi et al., 2016). GA-based wavelength selection has been successfully applied to improve SSC prediction in multiple studies (Roger and Bellon-Maurel, 2000; Ouyang et al., 2012; Almoujahed et al., 2025).

The objectives of this study were threefold: (i) to construct tomato SSC prediction models using linearly transformed spectral indices optimized by a GA and to compare their performance with models based on conventional two-band indices and PLS; (ii) to assess the extent to which model performance depends on tomato type; and (iii) to generate a virtual spectral reflectance landscape to visualize the distribution patterns of sensitive wavelength combinations. This study proposes a GA-optimized framework based on linearly transformed spectral indices for robust estimation of tomato SSC across multiple tomato types, providing a practical tool for harvest and postharvest quality management.

## 2 Materials and methods

### 2.1 Samples

Thirteen fresh-market tomato cultivars were selected: ‘Gaofen No.1’ (a locally recommended cultivar), ‘Mingzhi’, ‘FQ581’, ‘HTO-32’, ‘HTO-40’, ‘HTO-41’, ‘HTO-44’, ‘HTO-47’, ‘Gaotianfen No.7’, ‘C575’, ‘HTO-19’, ‘HHTO-35’, and ‘HHTO-36’. All plants were cultivated in multi-span plastic greenhouse structures with steel frames at the Vegetable Research Institute of Huai’an, Jiangsu Province, China. Seeds were sown on January 8, 2025, and transplanted on March 20, 2025. A randomized block design was adopted. Each cultivar was planted in a single plot measuring 1.8 m × 4.0 m. Plants were grown on flat beds with trellising, with 40 cm spacing between plants and 60 cm between rows. Irrigation and weed control followed standard local management practices. The basal fertilization consisted of 600 kg/ha compound fertilizer (N-P_2_O_5_-K_2_O = 15-15-15; Anhui Huilong Zhongcheng Technology Co., Ltd., Hefei, Anhui, China) and 7500 kg/ha organic fertilizer containing *Trichoderma* spp. (Huai’an Chaimihe Agricultural Technology Co., Ltd., Huai’an, Jiangsu, China). Additional fertilization included 225 kg/ha compound fertilizer (N-P_2_O_5_-K_2_O = 17-17-17; Shandong Runhe Fertilizer Co., Ltd., Jining, Shandong, China) applied at the fruit enlargement stage of the first truss, 90 kg/ha potassium fulvic acid water-soluble fertilizer (Shandong Runhe Fertilizer Co., Ltd.) applied after fruit set of the second truss, and 75 kg/ha potassium-rich water-soluble fertilizer (Shandong Runhe Fertilizer Co., Ltd.) applied after every one to two harvests. In addition, foliar sprays of KH_2_PO_4_ and calcium fertilizer (Anhui Ruilin Modern Agricultural Technology Co., Ltd., Hefei, Anhui, China) were applied once to prevent blossom-end rot.

Fruits were harvested at the full-ripe stage. Six plants were randomly selected from each plot. For large-fruited tomatoes, one to two fruits were collected per plant; for medium-sized and cherry tomatoes, two to three fruits were collected per plant. Cracked fruits were removed after harvest.

### 2.2 Spectral reflectance and SSC measurement

**Figure 1:**
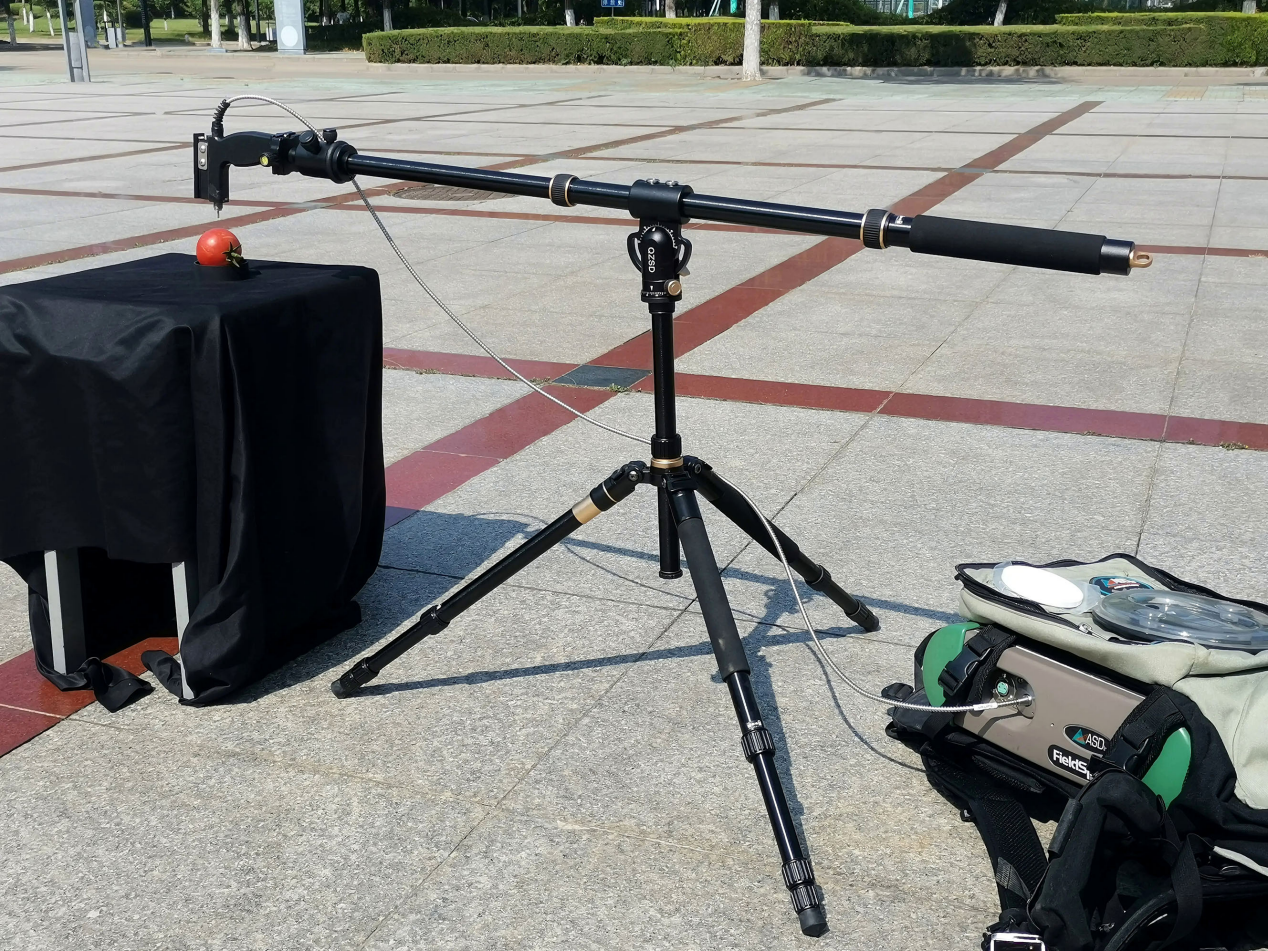
*In situ* measurement of tomato fruit spectra.

Spectral reflectance was measured on the day of harvest or the following day using a FieldSpec^®^ 4 spectroradiometer (ASD Inc., Alexandria, VA, USA). The instrument was calibrated using a white reference panel, and the fiber-optic probe was positioned vertically above the equatorial region of the fruit (**Figure 1**). Ten consecutive measurements were taken per fruit and averaged to obtain a single reflectance spectrum. The fruit was placed on a black concave tray to minimize movement during measurement. All spectral data were collected between 10:00 and 14:00 under clear-sky conditions.

Within one hour after spectral measurement, each fruit was juiced, and SSC (°Brix) was determined using a refractometer. Each fruit was measured three times, and the average was calculated. To evaluate model robustness, the dataset was randomly partitioned 10 times, generating ten pairs of modeling and validation subsets. In each iteration, 75% of the samples were used for model development, and the remaining 25% were reserved for independent validation. These ten randomized splits were denoted as Dataset 1 through Dataset 10.

### 2.3 Spectral data preprocessing

The FieldSpec^®^ 4 spectroradiometer covers a spectral range of 350–2500 nm, including visible, near-infrared, and shortwave infrared regions. Due to the high correlations among adjacent bands, raw data were averaged every 10 bands, and the midpoint wavelength was recorded. For example, the mean reflectance at 350–359 nm was assigned to 355 nm. Spectral regions strongly affected by water absorption (1345–1425, 1795–1975, and 2345–2500 nm) were removed.

### 2.4 Construction of linearly transformed spectral indices–based SSC prediction models

Reflectance values at selected bands were subjected to linear transformation and used to construct new two-band spectral indices analogous to the DSI, NDSI, and RSI. These were denoted as ltDSI_λ1,λ2_, ltNDSI_λ1,λ2_ and ltRSI_λ1,λ2_, respectively (where “lt” denotes linearly transformed reflectance). The two wavelengths are represented by λ1 and λ2, and their order is unrestricted. The indices were defined as shown in Equations 1–3:

(1) $${\text{ltDSI}}_{\lambda 1,\lambda 2}=k_{1}\cdot R_{\lambda 1}-k_{2}\cdot R_{\lambda 2}+b$$

(2) $${\text{ltNDSI}}_{\lambda 1,\lambda 2}=\frac{k_{1}\cdot R_{\lambda 1}-k_{2}\cdot R_{\lambda 2}+b_{1}}{k_{1}\cdot R_{\lambda 1}+k_{2}\cdot R_{\lambda 2}+b_{2}}$$

(3) $${\text{ltRSI}}_{\lambda 1,\lambda 2}=\frac{k_{1}\cdot R_{\lambda 1}+b_{1}}{k_{2}\cdot R_{\lambda 2}+b_{2}}$$

where *R*_λ_ is the reflectance at wavelength λ, and *k*_1_, *k*_2_, *b*, *b*_1_, and *b*_2_ are the coefficients to be optimized. Linear regression models based on these indices were constructed to estimate SSC, and their coefficients of determination (*R*^2^) were calculated.

Model construction based on ltDSI_λ1,λ2_, ltNDSI_λ1,λ2_, and ltRSI_λ1,λ2_ required the simultaneous optimization of both band selection and transformation coefficients, representing a high-dimensional, non-deterministic polynomial-time (NP-hard) problem. To address this, a GA was employed in which each candidate solution was represented as a chromosome, and genetic operators, including recombination (crossover), mutation, fitness evaluation, selection, and inter-population migration, were iteratively applied to the population of chromosomes.

For ltDSI_λ1,λ2_, the coefficients *k*_1_, *k*_2_, and *b* were optimized, as represented by the chromosome structure in **Supplementary Figure S1A**. For ltNDSI_λ1,λ2_ and ltRSI_λ1,λ2_, the coefficients *k*_1_, *k*_2_, *b*_1_, and *b*_2_ were optimized, as illustrated in **Supplementary Figure S1B**. The model *R*^2^ was used as the fitness function.

The GA adopted a multi-population strategy with 10 subpopulations of 100 individuals each, a migration rate of 0.2, and migration every 10 generations. The maximum generation number (MaxGen) was set to 50, and the initial crossover rate (XOVR) and mutation rate (MUTR) were set to 0.7 and 0.2, respectively, and updated at each generation based on the following rules, as shown in Equations 4, 5:

(4) $$XOVR=XOVR-\frac{0.7-0.3}{MaxGen}$$

(5) $$MUTR=MUTR-\frac{0.2-0.005}{MaxGen}$$

This adaptive adjustment strategy was designed to maintain population diversity and improve optimization performance. The SSC prediction models were constructed and validated using the dataset described in Section 2.2.

### 2.5 Construction of conventional two-band spectral indices–based SSC prediction models

Conventional two-band spectral indices are derived by applying basic arithmetic operations to reflectance values. In this study, three such indices, DSI, NDSI, and RSI, were constructed. For each index, all possible band-pair combinations were computed, and the corresponding *R*² with SSC were evaluated. The band pairs yielding the highest *R*² values were then selected to develop the SSC prediction model. For arbitrary band pairs (λ1, λ2; λ1 < λ2), the indices were calculated as follows, as shown in Equations 6–8:

(6) $${\text{DSI}}_{\lambda 1,\lambda 2}=R_{\lambda 1}-R_{\lambda 2}$$

(7) $${\text{NDSI}}_{\lambda 1,\lambda 2}=\frac{R_{\lambda 1}-R_{\lambda 2}}{R_{\lambda 1}+R_{\lambda 2}}$$

(8) $${\text{RSI}}_{\lambda 1,\lambda 2}=\frac{R_{\lambda 1}}{R_{\lambda 2}}$$

where *R*_λ_ is the reflectance at wavelength λ. Linear regression models were constructed to estimate SSC, and the *R*^2^ was calculated. The SSC prediction models were constructed and validated using the dataset 1 described in Section 2.2.

### 2.6 Construction of full-spectrum PLS SSC prediction model

Partial least squares (PLS) regression is a widely used multivariate modeling technique, particularly suitable for datasets with many predictors, strong collinearity, noise, or limited sample size. It has been extensively applied to Vis/NIR spectroscopy for predicting fruit quality attributes (Zheng et al., 2024). In this study, a full-spectrum PLS model was developed to predict SSC, serving as a baseline for evaluating the performance of the models constructed in Section 2.4. During model development, the optimal number of latent variables (LVs) was determined at the point where the explained variance approached 100% and the residual variance approached zero. The calibration and validation of the PLS model were performed using the dataset described in Section 2.2.

### 2.7 Model evaluation

The performance of the models was assessed using the following statistical metrics: the coefficients of determination for calibration and prediction ( $R_{c}^{2}$ and $R_{p}^{2}$), the root mean square errors of calibration and prediction (RMSEC and RMSEP), and the mean relative error (MRE). A higher *R*² indicates better predictive ability, whereas lower *RMSE* and *MRE* values are desirable (Li H. et al., 2024). In addition, the coefficient of variation (CV) was used to quantify the variability in SSC across different tomato cultivars. These metrics were calculated as, as shown in Equations 9–12:

(9) $$R^{2}=1-\frac{\sum_{i=1}^{n}{\left(y_{i}-{\hat{y}}_{i}\right)}^{2}}{\sum_{i=1}^{n}{\left(y_{i}-{\bar{y}}_{i}\right)}^{2}}$$

(10) $$RMSE=\sqrt{\frac{1}{n}\sum_{i=1}^{n}{\left(y_{i}-{\hat{y}}_{i}\right)}^{2}}$$

(11) $$MRE=\frac{1}{n}\sum_{i=1}^{n}\left|\frac{{\hat{y}}_{i}-y_{i}}{y_{i}}\right|\times 100$$

(12) $$CV=\frac{1}{\bar{x}}\sqrt{\frac{\sum_{i=1}^{n}{\left(y_{i}-\bar{y}\right)}^{2}}{n-1}}\times 100$$

where $y_{i}$ is the observed value; $\bar{y}$ is the mean of the observed values; ${\hat{y}}_{i}$ is the predicted value; and n is the sample size.

## 3 Results

### 3.1 Variability of tomato SSC

The tomato fruits used in this study were classified into four types: large red, medium red, red cherry, and yellow cherry. A total of 152 samples were analyzed, with SSC values ranging from 3.0 to 8.5°Brix (**Table 1**). Pronounced differences in SSC distribution were observed among the four tomato types. The five large-red varieties exhibited relatively low SSC values, suggesting lower sweetness levels. Their SSC dispersion was limited, with most varieties showing CVs of approximately 4%, except for Mingzhi, which showed a slightly higher CV exceeding 6%. The medium-red group consisted of three varieties and exhibited greater SSC heterogeneity. In particular, HTO-44 showed substantial variability, with a CV exceeding 15%. The cherry-type tomatoes included five varieties and were characterized by generally higher SSC values. However, their variability differed among varieties: three showed moderate dispersion with CVs close to 5%, whereas the remaining two exhibited higher variability, with CVs equal to or greater than 12%. Overall, the wide SSC range and the pronounced variability observed across tomato types and varieties provide a robust data basis for developing reliable SSC prediction models, which is beneficial for improving both model accuracy and generalization performance.

**Table 1:** Statistical summary of tomato sample types and their soluble solids content.

Variety	Range (°Brix)	No. of samples	Mean (°Brix)	Coefficient of variation (%)	Tomato type
Gaofen No.1	4.0–4.7	11	4.2	4.4	Large red
Mingzhi	3.9–4.7	9	4.3	6.1	Large red
FQ581	3.7–4.3	9	4.1	4.0	Large red
HTO-32	3.8–4.2	8	4.0	3.6	Large red
HTO-40	4.6–5.2	6	4.9	4.0	Large red
HTO-41	4.8–6.8	15	5.7	10.7	Medium red
HTO-44	3.0–5.1	13	4.2	15.1	Medium red
C575	3.2–4.0	12	3.6	6.5	Medium red
Gaotianfen No.7	6.5–7.5	15	7.0	4.9	Red cherry
HTO-47	5.1–7.5	16	6.4	13.6	Red cherry
HTO-19	6.8–7.7	12	7.2	4.3	Red cherry
HHTO-35	6.2–7.7	10	7.0	5.8	Yellow cherry
HHTO-36	6.0–8.5	16	7.2	12.0	Yellow cherry

### 3.2 Spectral reflectance characteristics of different tomato types

**Figure 2:**
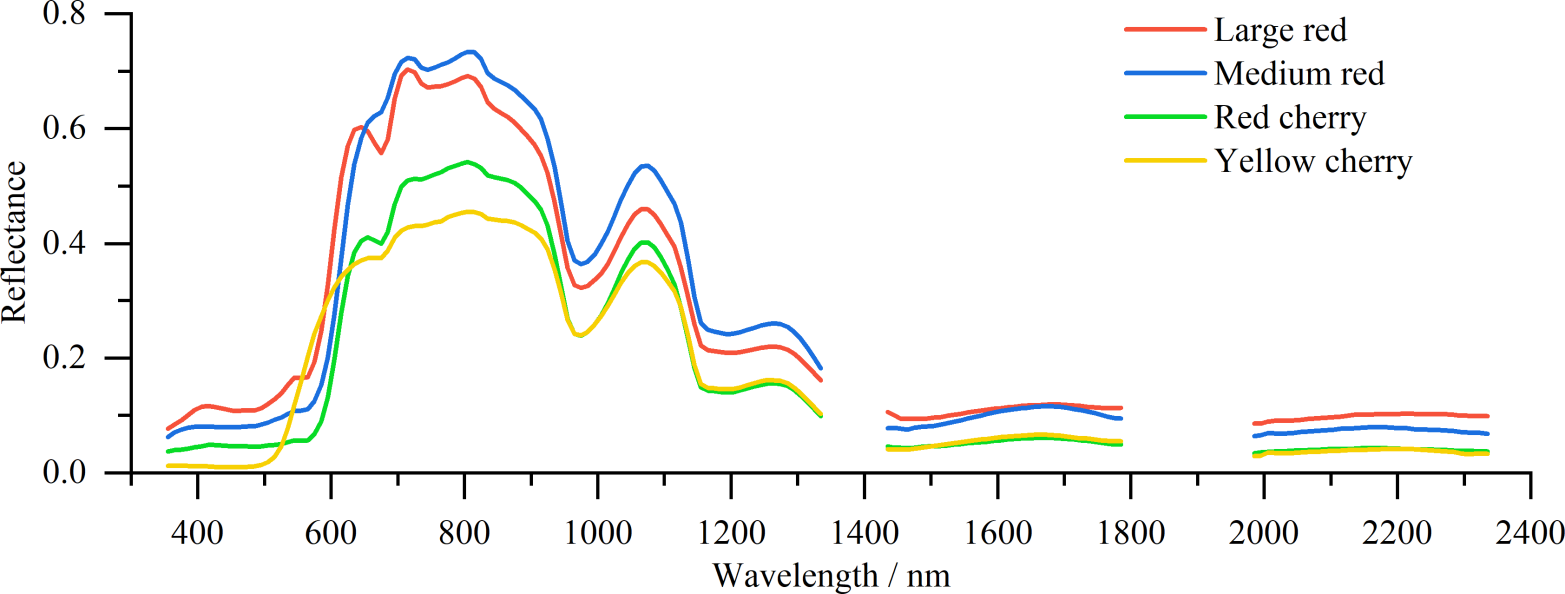
Spectral reflectance curves of different tomato types (large red, medium red, red cherry, and yellow cherry) across the measured wavelength range.

**Figure 2** presents the spectral reflectance curves for the four tomato types. In the visible region below 600 nm, reflectance was generally low for all samples. A distinct red-edge feature appeared near 600 nm. Compared with the other tomato types, yellow cherry tomatoes exhibited a noticeable blue shift in the red-edge position. In the 600–1300 nm region, multiple reflectance peaks and troughs were observed, indicating rich spectral features that are potentially informative for SSC modelling. These reflectance features were sensitive to tomato type. In particular, the troughs allowed discrimination among large, medium, and cherry tomatoes, whereas differentiation between red and yellow cherry types was limited due to substantial overlap of their spectral curves. By contrast, the 1400–2400 nm region exhibited relatively flat reflectance patterns, with nearly overlapping curves for red and yellow cherry tomatoes. This suggests that this spectral range provides limited discriminatory information for differentiating tomato types (**Figure 2**).

### 3.3 GA-optimized linearly transformed reflectance spectral indices for prediction models

**Figure 3:**
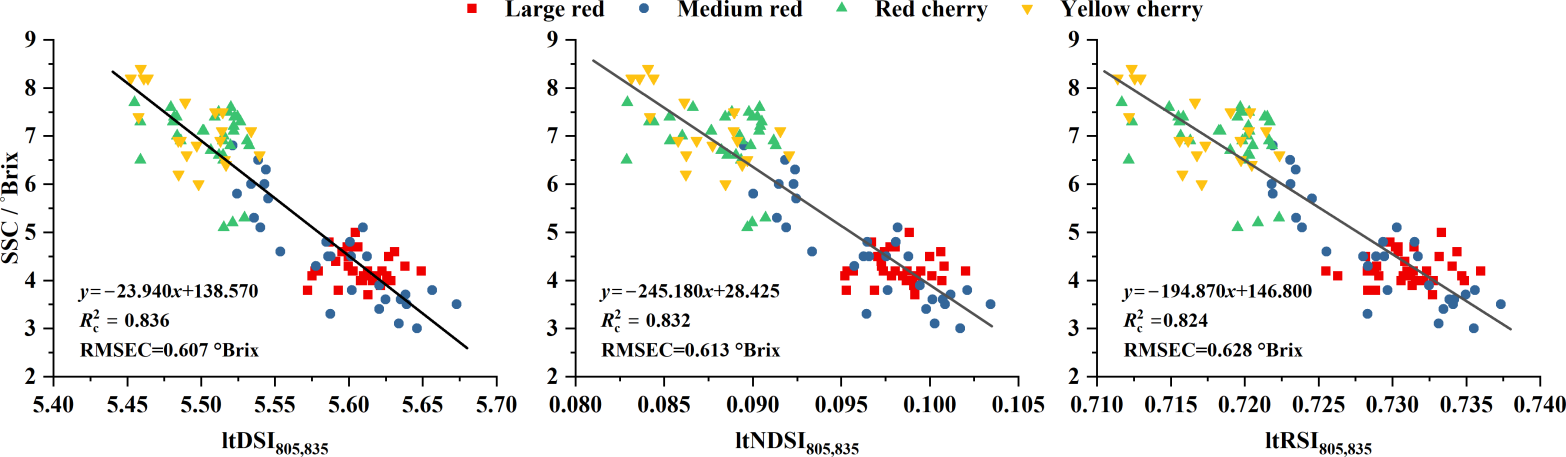
Prediction performance of GA-optimized spectral indices (ltDSI, ltNDSI, and ltRSI) for tomato SSC using the calibration Dataset 1.

Using the ten datasets described in Section 2.2, GA was applied to construct the ltDSI, ltNDSI, and ltRSI indices. These indices were then used to develop SSC prediction models, and the calibration performance is summarized in **Supplementary Table S2**. For Dataset 1, the three spectral indices are given in Equations 13–15), and their predictive performance is shown in **Figure 3**. As shown in **Supplementary Table S2**, only the indices derived from Dataset 8 selected different wavelength pairs, and their $R_{c}^{2}$ values were relatively low. For Dataset 5, ltDSI, ltNDSI, and ltRSI all selected the same pair of wavelengths (805 and 845 nm). For the remaining eight datasets, all three indices consistently selected 805 and 835 nm. Overall, these wavelength pairs yielded high $R_{c}^{2}$ values, indicating that 805 and 835 nm are sensitive wavelengths for SSC prediction.

**Figure 4:**
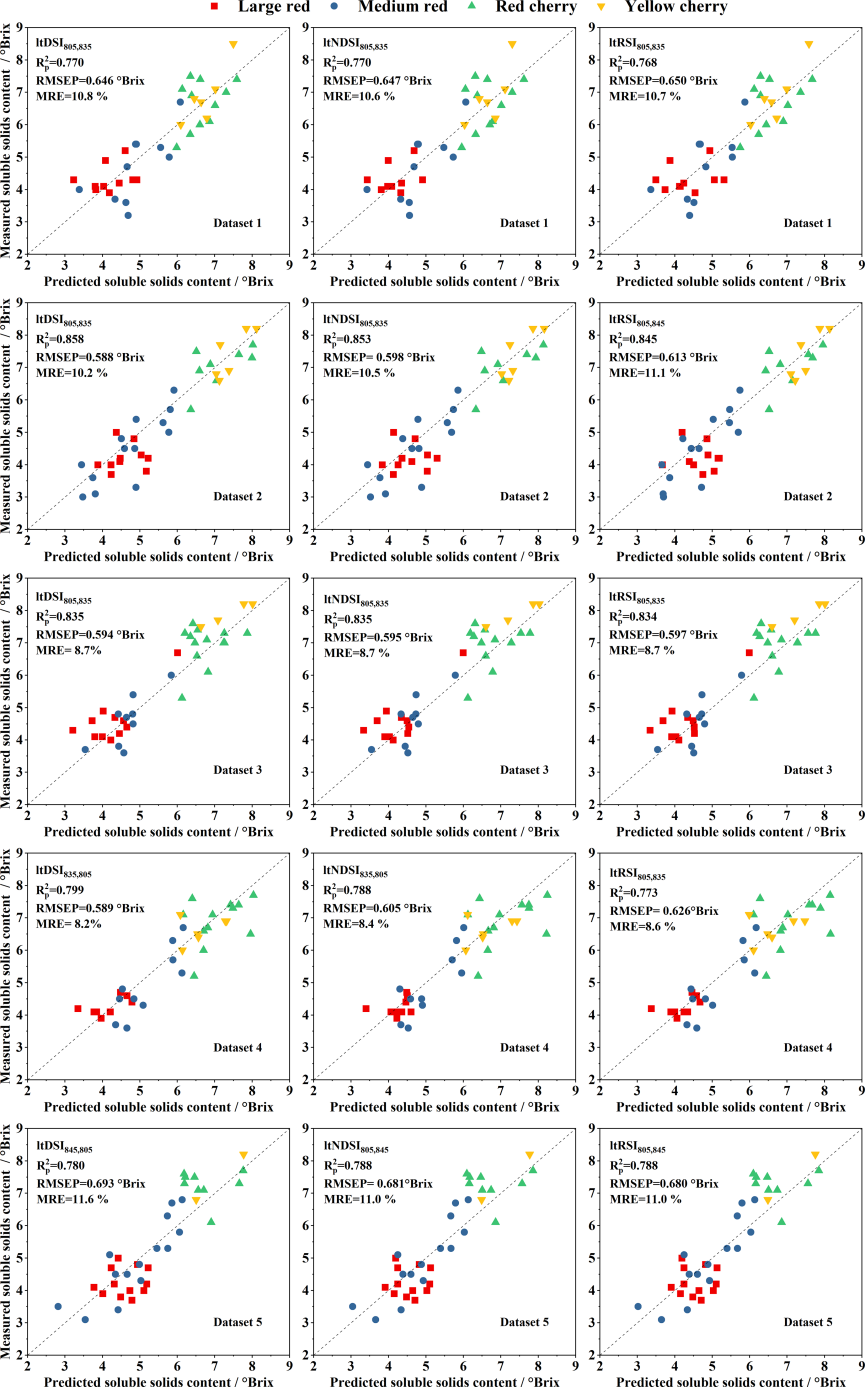
Relationships between GA-optimized predicted and measured SSC values for the validation Datasets 1–5, with the dashed line indicating the 1:1 reference.

The validation performance for Datasets 1–5 is presented in **Figure 4**, where all data points cluster closely around the 1:1 line. The corresponding validation results for Datasets 6–10 are provided in **Supplementary Figure S3**, showing similar performance trends. Across all datasets, the models achieved $R_{p}^{2}$ values of approximately 0.8, RMSEP values around 0.6°Brix, and MRE values near 10%. For a given dataset, the $R_{p}^{2}$, RMSEP, and MRE of ltDSI, ltNDSI, and ltRSI were highly similar, except for Dataset 8. As illustrated in **Figure 4**; **Supplementary Figure S3**, none of the models exhibited sensitivity to tomato variety, demonstrating strong generalization capability.

(13) $${\text{ltDSI}}_{805,835}=4.772R_{805}-4.773R_{835}+5.410$$

(14) $${\text{ltNDSI}}_{805,835}=\frac{-7.202R_{805}+5.974R_{835}-0.330}{-7.202R_{805}-5.974R_{835}-5.639}$$

(15) $${\text{ltRSI}}_{805,835}=\frac{2.717R_{805}+1.378}{3.808R_{835}+1.979}$$

Where *R*_805_ and *R*_835_ denote the reflectance at 805 nm and 835 nm, respectively.

**Figure 5:**
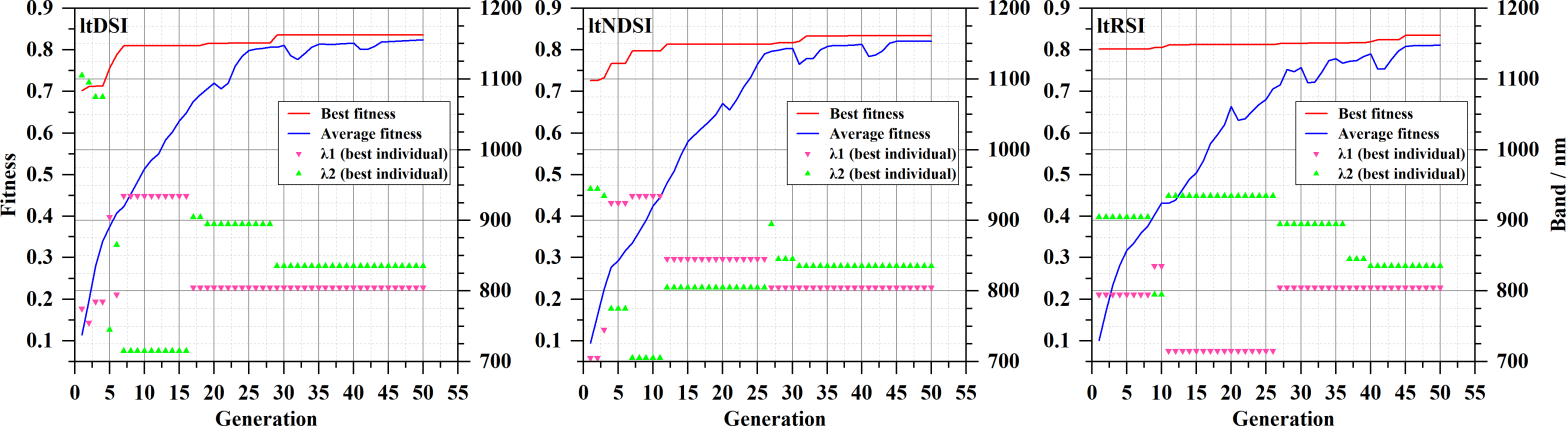
Evolution of the best fitness ( $R_{c}^{2}$), average population fitness, and corresponding wavelength combinations (λ1, λ2) during GA optimization of spectral indices.

**Figure 5** illustrates the evolutionary process of the GA in constructing spectral indices using Dataset 1. It shows the fitness of the best individual (defined as $R_{c}^{2}$ in this study), the corresponding band combinations (λ1 and λ2), and the mean fitness of the population. When the GA was used to optimize ltDSI, ltNDSI, and ltRSI, different band combinations were selected at the initial stage. After approximately 40 generations, all three indices converged to the same band pair of 805 and 835 nm. It is worth noting that, during the evolutionary process, the GA also identified other band combinations that achieved high predictive performance, with $R_{c}^{2}$ values exceeding 0.8. Examples of these optimized indices include ltDSI_935,715_, ltDSI_805,905_, and ltDSI_805,895_; ltNDSI_935,705_, ltNDSI_845,805_, ltNDSI_805,895_, and ltNDSI_805,845_; as well as ltRSI_835,795_, ltRSI_715,935_, and ltRSI_805,895_.

**Figure 6:**
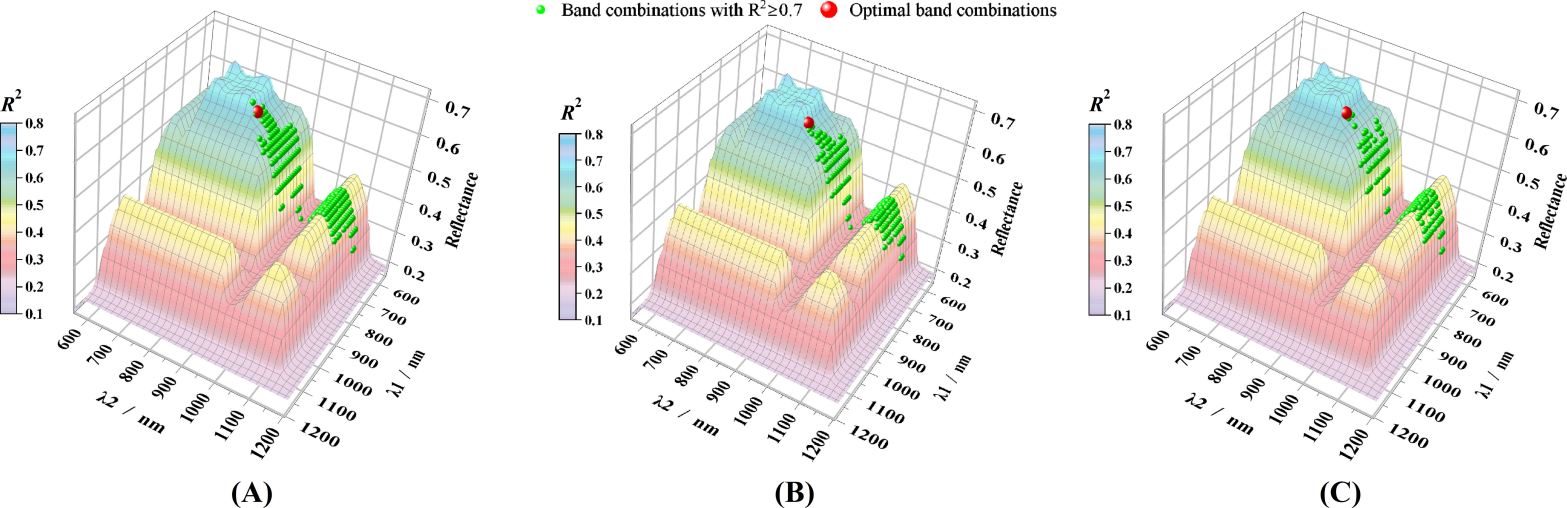
Virtual spectral reflectance landscape illustrating the distribution of SSC-sensitive wavelength combinations ( $R_{c}^{2}$ ≥ 0.7) identified after 10 GA generations for **(A)** ltDSI, **(B)** ltNDSI, and **(C)** ltRSI.

A virtual reflectance landscape was constructed using the spectral reflectance of large red tomatoes within the 555–1205 nm range to further illustrate spectral sensitivity (**Figure 2**). The side projection of this landscape corresponds to the spectral reflectance curve as a function of wavelength. Both the X- and Y-axes represent wavelengths from 555 to 1205 nm, and the Z-axis represents the reflectance values, defined as Z(x,y)=min(R(x),R(y)), where R(x) and R(y) are reflectance values at wavelengths *x* and *y*, respectively, and min() denotes the minimum operator. The number of generations was limited to 10 when constructing GA-optimized ltDSI, ltNDSI, and ltRSI models. At the end of evolution, band combinations with R^2^ ≥ 0.7 were plotted on the surface of the virtual landscape as green spheres (**Figure 6**). The optimal band pairs obtained at the end of GA evolution (**Figure 5**) were also mapped in the landscape as red spheres. As shown in **Figure 6**, the green spheres were concentrated in a small region of the landscape, with λ1 approximately 700–850 nm and λ2 820–1150 nm.

### 3.4 Two-band conventional spectral index–based prediction models

**Figure 7:**
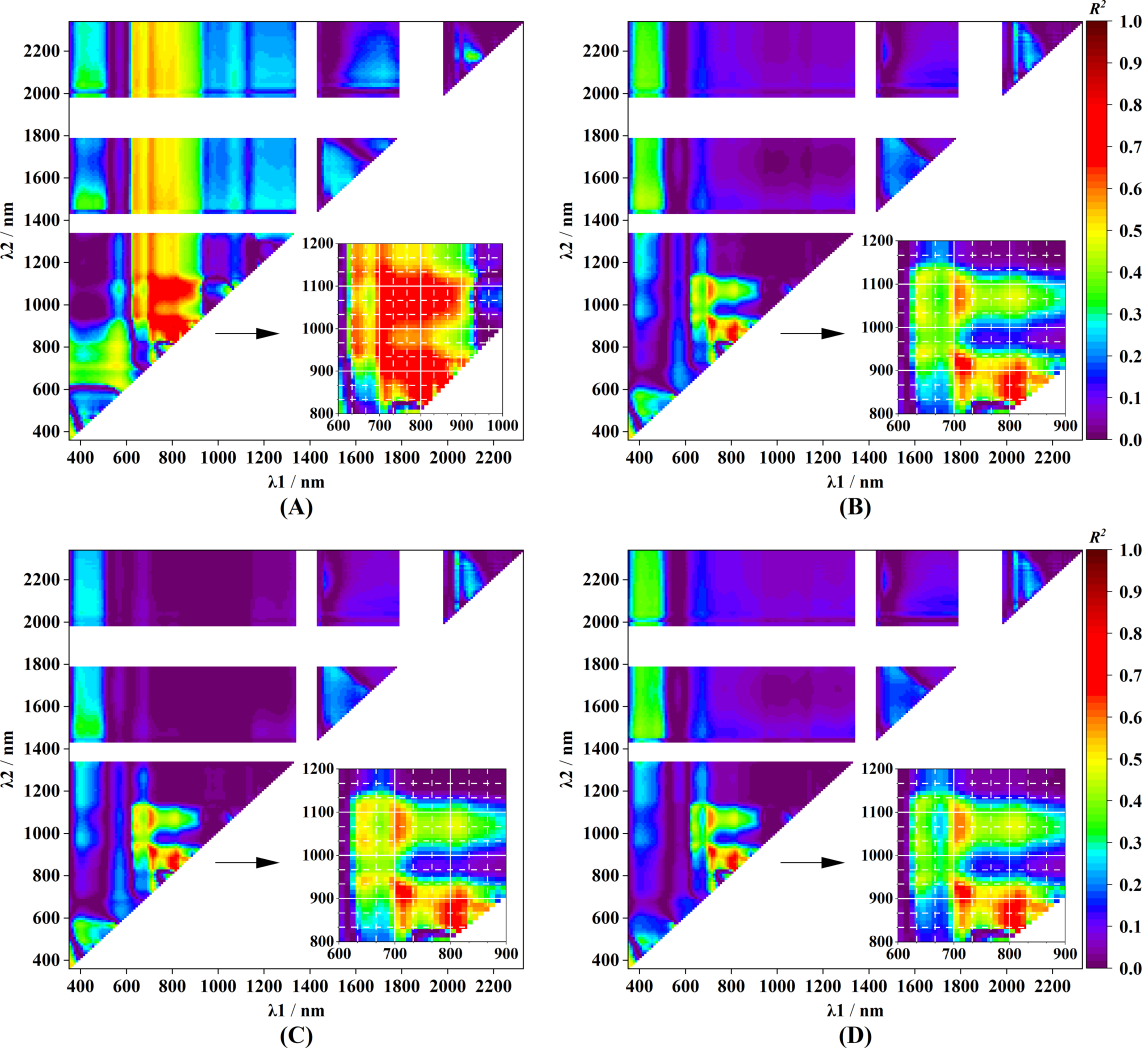
Heat maps of $R_{c}^{2}$ values between SSC and conventional two-band spectral indices across all wavelength combinations: **(A)** DSI_λ1,λ2_, **(B)** NDSI_λ1,λ2_, **(C)** RSI_λ1,λ2_, and **(D)** RSI_λ2,λ1_.

**Figure 7** shows the distribution of the *R*^2^ values for SSC prediction using conventional two-band spectral indices, where red areas indicate *R*^2^ > 0.6. The red area was much larger for DSI_λ1,λ2_ (**Figure 7A**) than for NDSI_λ1,λ2_ (**Figure 7B**), RSI_λ1,λ2_ (**Figure 7C**), and RSI_λ2,λ1_ (**Figure 7D**). For SSC prediction, when λ1 ranged from 700 to 900 nm and λ2 ranged from 830 to 1130 nm, multiple band combinations achieved satisfactory performance, with *R*^2^ values between 0.65 and 0.82. Additionally, when λ1 was 600–800 nm and λ2 was > 1200 nm, DSI achieved a relatively higher prediction accuracy, with *R*^2^ values between 0.50 and 0.62. The highest *R*^2^ values (>0.65) of NDSI_λ1,λ2_ were concentrated in narrow ranges, with λ1 ranging from 700 to 830 nm and λ2 ranging from 830 to 930 nm, yielding a maximum *R*^2^ of 0.73. The distribution of RSI was similar to that of DSI, with overlapping sensitive regions (**Figure 7**).

**Figure 8:**
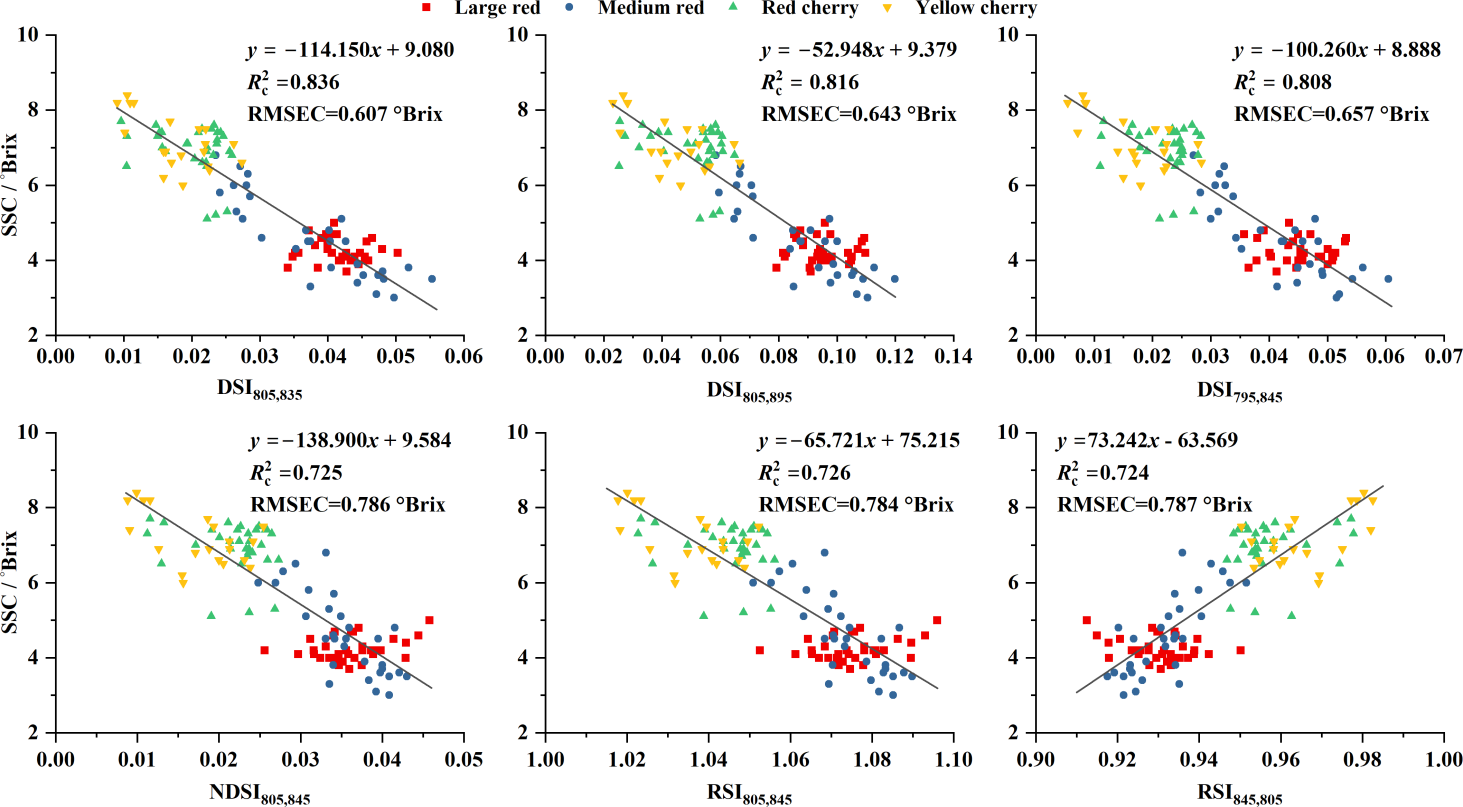
Prediction performance of selected conventional spectral indices for tomato SSC on Validation Dataset 1.

Based on **Figure 7**, the best-performing conventional two-band spectral indices were selected to construct SSC prediction models. For DSI, the indices DSI_805,835_, DSI_805,895_, and DSI_795,845_ were identified, whereas NDSI_805,845_, RSI_805,845_, and RSI_845,805_ were selected for the other index types. As shown in **Figure 8**, DSI_805,835_, DSI_805,895_, DSI_795,845_, NDSI_805,845_, and RSI_805,845_ exhibited negative correlations with SSC, whereas RSI_845,805_ showed a positive correlation. Among all conventional indices, DSI-based models achieved the highest calibration performance, with $R_{c}^{2}$ values of 0.836, 0.816, and 0.808, while the remaining indices yielded only marginally higher values than 0.720. Notably, the distribution pattern of data points corresponding to large red tomatoes deviated from the overall trend in models based on NDSI and RSI, indicating a pronounced sensitivity of these models to fruit type. This type-dependent behavior suggests limited generalization when conventional indices are applied to heterogeneous datasets. The validation results further confirmed the superior performance of DSI-based models (**Supplementary Figure S4**), which exhibited lower RMSEP values (0.64–0.66°Brix) and a lower MRE (approximately 10.8%) compared with NDSI- and RSI-based models (RMSEP > 0.71°Brix and MRE > 11.0%).

We noticed that two subplots in **Figure 8**, corresponding to SSC prediction using DSI_805,835_ and DSI_805,895_, exhibit partial visual similarity. This similarity primarily arises from the strong linear relationship between the reflectance at 835 nm and 895 nm (R^2^ = 0.985), which in turn leads to a strong linear correlation between the corresponding spectral indices DSI_805, 835_ and DSI_805, 895_ (R^2^ = 0.989), as illustrated in **Supplementary Figure S5**. In addition, the subplots based on NDSI_805,845_ and RSI_805,845_ show an even higher degree of global similarity in **Figure 8**. This can be attributed to the strong linear relationship between NDSI and RSI derived from the same pair of spectral bands in the present dataset. Let the reflectance at two wavelengths be denoted as R_1_ and R_2_. NDSI and RSI are defined as $NDSI=(R_{1}-R_{2})/(R_{1}+R_{2})$ and $RSI=R_{1}/R_{2}$, respectively. By rearranging these expressions, the following relationship can be obtained: 1/NDSI=1+2/(RSI−1). When RSI is close to 1, |RSI−1|≪2, and NDSI can be approximated as NDSI≈0.5RSI−0.5, indicating a strong linear dependence between the two indices. As shown in **Figure 8**, RSI_805,845_ values in this study mainly range from 1.0 to 1.1 and are therefore very close to 1, which explains the strong linear relationship between NDSI and RSI observed in our results. This is further supported by the highly similar distribution patterns in **Figures 7B, C**. Similar behavior has also been reported by Tanaka et al. (2015), who observed a very high degree of similarity between NDSI- and RSI-based models for leaf area index estimation (see **Figure 3** in their study).

### 3.5 Full-spectrum PLS models for SSC prediction

Using PLS, full-spectrum SSC prediction models were constructed based on the ten datasets described in Section 2.2, and their predictive performance is summarized in **Table 2**. These models were used as benchmark models for comparison with the GA-optimized linearly transformed reflectance spectral index–based prediction models. For all datasets, the optimal number of LVs was three. Across the ten datasets, the $R_{p}^{2}$ values ranged from 0.539 to 0.767, with a mean of 0.668. The RMSEP varied between 0.720 and 1.012°Brix, with an average of 0.822°Brix. The MRE ranged from 9.7% to 14.3%, with a mean of 12.2%. Overall, the predictive accuracy of the full-spectrum PLS models was moderate and showed noticeable variation among datasets. These differences can be attributed to variations in sample composition between the calibration and validation sets, resulting from the random dataset partitioning strategy.

**Table 2:** Prediction performance of full-spectrum partial least squares (PLS) models for SSC using different calibration and validation dataset partitions.

Dataset	Number of LVs	$R_{c}^{2}$	$R_{p}^{2}$	RMSEC (°Brix)	RMSEP (°Brix)	MRE (%)
1	3	0.741	0.593	0.763	0.861	12.5
2	3	0.670	0.767	0.821	0.757	12.8
3	3	0.680	0.751	0.824	0.742	9.7
4	3	0.726	0.615	0.788	0.819	11.0
5	3	0.677	0.600	0.826	0.942	14.3
6	3	0.670	0.683	0.853	0.786	10.4
7	3	0.735	0.642	0.766	0.826	12.8
8	3	0.693	0.539	0.806	1.012	14.6
9	3	0.709	0.734	0.791	0.754	12.9
10	3	0.682	0.760	0.824	0.720	10.5

To further compare model performance under identical data conditions, paired *t*-tests were conducted using the $R_{p}^{2}$, RMSEP, and MRE values of models developed from the same datasets (**Table 2**, **Figure 4**; **Supplementary Figure S3**). The results indicated that SSC prediction models based on the ltDSI significantly outperformed the corresponding PLS models, with *p*-values of 0.001, 3.30 ×10^-4^, and 0.004 for $R_{p}^{2}$, RMSEP, and MRE, respectively (*p* < 0.01). Similarly, models based on ltNDSI (*p* = 4.65 × 10^-4^, 1.63 × 10^-4^, and 0.002) and ltRSI (*p* = 0.001, 4.52 × 10^-4^, and 0.004) also showed significantly superior predictive performance compared with the PLS models. These results demonstrate that the GA-optimized spectral index–based models provide more accurate SSC predictions than full-spectrum PLS models under the same dataset conditions.

## 4 Discussion

### 4.1 Spectral mechanisms underlying SSC prediction

The consistent selection of wavelength pairs around 805 and 835 nm across multiple datasets suggests that this spectral region is closely associated with tomato SSC. Fruit SSC is mainly determined by soluble sugars, including glucose, fructose, and sucrose (McGlone and Kawano, 1998; Zhou et al., 2023), with minor contributions from organic acids (Zhan et al., 2023; Cai et al., 2024). In tomatoes, glucose and fructose are the dominant sugars, whereas citric acid and malic acid are the primary organic acids (Ibáñez et al., 2019). Glucose and fructose are an aldose and a ketose, respectively, but they share very similar chemical structures (Qi and Tester, 2019). Each molecule contains multiple hydroxyl (O–H) groups and one carbonyl (C=O) group, which makes them spectrally difficult to distinguish in intact fruit. As a result, SSC-related spectral information in the near-infrared region mainly reflects the collective absorption and scattering effects of these functional groups rather than individual sugar species.

Previous studies have reported that absorption features near 800 nm are associated with combination and overtone vibrations of O–H bonds (Afonso et al., 2022). Given the abundance of hydroxyl groups in glucose and fructose, this spectral region is likely sensitive to changes in soluble sugar concentration. This provides a plausible optical explanation for the consistent selection of 805 and 835 nm by the GA in SSC prediction. Notably, the sensitive wavelengths identified in this study fall within the spectral range (780–980 nm) previously reported for tomato SSC prediction (Peiris et al., 1998) and overlap with the optimal wavelength range identified for pear SSC estimation (700–930 nm) (Choi et al., 2017). In contrast, these wavelengths differ substantially from those reported for kiwifruit SSC prediction, which are mainly concentrated between 900 and 930 nm (McGlone and Kawano, 1998). This discrepancy may be attributed to differences in SSC composition, as sucrose accounts for a higher proportion of total soluble sugars in kiwifruit compared with tomatoes. The stability of these wavelengths across datasets further indicates that they capture robust SSC-related spectral responses rather than cultivar-specific features.

In addition, other wavelength combinations identified during the GA evolutionary process (**Figure 5**) may be related to higher-order overtone vibrations of O–H and C–H bonds (Afonso et al., 2022; Zheng et al., 2024). This suggests that incorporating additional spectral bands may further improve SSC prediction performance, which warrants investigation in future studies.

### 4.2 Advantages of GA-optimized linearly transformed spectral indices

Conventional two-band spectral indices, such as DSI, NDSI, and RSI, are typically constructed using fixed mathematical formulations and predefined wavelength combinations (Kasimati et al., 2022; Wang Q. et al., 2022). Although these indices are simple and computationally efficient, their rigid structure limits adaptability when applied to heterogeneous datasets involving multiple cultivars or varying spectral baselines. As a result, their predictive performance and generalization ability are often sensitive to dataset composition, as illustrated in **Figure 8**.

In contrast, the GA-optimized linearly transformed spectral indices proposed in this study simultaneously optimize both wavelength selection and transformation coefficients. This integrated optimization enables the indices to adapt to variations in spectral magnitude and baseline shifts caused by cultivar differences and measurement conditions. Compared with approaches that first select wavelengths and subsequently construct prediction models (Ouyang et al., 2012; González-Flor et al., 2014), the proposed strategy offers clear advantages in terms of both prediction accuracy and model generalization. The reduced cultivar sensitivity observed across multiple independently generated datasets indicates that jointly optimizing index structure and parameters plays a critical role in enhancing model robustness and transferability. Rather than relying on fixed index formulations, the improved performance appears to stem from increased model flexibility, which allows the indices to accommodate biological and spectral variability more effectively. Similar challenges associated with cultivar sensitivity have been reported in previous studies. For example, Ibáñez et al. (2019) demonstrated that SSC prediction performance based on diffuse reflectance near-infrared spectra varied substantially across tomato cultivars. Likewise, Kusumiyati et al. (2022) reported difficulties in developing a universal SSC prediction model for melon. In the present study, SSC prediction models based on conventional two-band spectral indices also exhibited pronounced cultivar sensitivity. In contrast, the GA-optimized models based on linearly transformed reflectance indices showed neither cultivar-specific nor tomato-type-specific sensitivity. This contrast suggests that cultivar dependence in SSC prediction may be strongly influenced by the structural form of the prediction model rather than by crop type alone.

Full-spectrum PLS models are widely used as benchmark approaches for SSC prediction because they can exploit information from the entire spectral range and effectively address multicollinearity (Zheng et al., 2024). However, in the present study, these models achieved only moderate predictive performance and were consistently outperformed by the GA-optimized ltDSI, ltNDSI, and ltRSI models, as confirmed by the paired t-test results in Section 3.5. Although substantially higher prediction accuracy has been reported in some previous studies, such as the PLS-based model developed by He et al. (2005) with an RMSEP of 0.16°Brix, the discrepancy is likely attributable to differences in dataset heterogeneity. Specifically, He et al. (2005) focused on a single tomato cultivar, whereas the present study included four tomato types comprising thirteen cultivars, introducing greater biological and spectral variability and imposing a more stringent test of model generalization.

Compared with both conventional two-band indices and full-spectrum PLS models, the GA-optimized spectral indices achieved comparable or higher prediction accuracy while maintaining a much simpler model structure. More importantly, their predictive performance remained stable across ten independently generated datasets, providing strong evidence of robustness and generalization. Unlike PLS models, which require the full spectral range and careful optimization of latent variables, the proposed indices rely on only two wavelengths and simple arithmetic operations. This simplicity makes them particularly suitable for implementation in low-cost multispectral sensors and real-time applications, without sacrificing predictive reliability. Given that agricultural products often exhibit substantial biological variability due to differences in cultivar, growing season, production year, and geographical origin (Tian et al., 2023), future studies should further evaluate the effects of seasonal, annual, and geographical variability to enhance model robustness and transferability.

### 4.3 Field-based spectral acquisition: advantages and challenges

As shown in **Figure 1**, spectral data in this study were acquired under outdoor conditions rather than in a fully controlled laboratory environment, such as measurements conducted in a darkroom (Huang et al., 2021; Zheng et al., 2024). Compared with laboratory-based measurements, field-based spectral acquisition inevitably introduces additional sources of variability, including fluctuations in ambient illumination and sensor–target geometry effects (Nicolai et al., 2007). These factors may increase spectral noise and complicate subsequent data processing and modeling.

Despite these challenges, field-based spectral acquisition also offers important advantages that help explain differences among reported SSC prediction performances in the literature. First, outdoor measurements more closely reflect realistic sensing conditions encountered in practical agricultural applications, thereby improving the ecological validity of the developed models. Second, the increased spectral variability inherent to field conditions provides a more stringent test of model robustness and generalization ability. Models that perform well under such conditions are therefore more likely to maintain reliable performance when deployed in real-world scenarios. In addition, outdoor measurements can avoid certain instabilities associated with artificial illumination sources commonly used in laboratory settings, such as lamp aging and power fluctuations (Cen and He, 2007).

It should also be noted that different spectral acquisition modes may contribute to discrepancies among studies. Transmittance-based measurements are often limited by the penetration depth of near-infrared radiation, particularly when applied to thick-peeled fruits, which restricts their ability to probe internal quality attributes (Todorova et al., 2024). In contrast, the present study employed reflectance-based spectral measurements under field conditions, which are less constrained by fruit thickness and are more suitable for non-destructive assessment of internal parameters in intact tomato fruits. This difference in measurement geometry provides a plausible explanation for variations in SSC prediction performance reported across studies using different acquisition strategies.

## 5 Conclusions

This study demonstrates that hyperspectral reflectance combined with GA optimization provides a robust and transferable framework for the non-destructive estimation of tomato SSC across multiple fruit types and cultivars. The linearly transformed reflectance–based spectral indices (ltDSI, ltNDSI, and ltRSI) consistently outperformed traditional spectral indices and full-spectrum PLS models and exhibited markedly reduced sensitivity to cultivar differences, underscoring the importance of index design in capturing physiologically relevant spectral information associated with SSC. These results indicate that carefully optimized spectral indices can effectively extract biologically meaningful signals related to sugar accumulation while remaining resilient to cultivar-induced spectral variability. Although this study focused on fully ripe fruits under specific measurement conditions, further validation across different developmental stages and field environments is needed to support broader physiological interpretation and practical application. Overall, this work provides a scalable, non-destructive approach for assessing fruit internal quality and contributes to the development of field-applicable phenotyping tools in horticultural crop research.

## Data Availability

The original contributions presented in the study are included in the article/**Supplementary Material**. Further inquiries can be directed to the corresponding author.
