# Supplementary material for: Novel estimation of tomato soluble solids content using linearly transformed reflectance-based spectral indices
Source: Front Plant Sci. 2026 Feb 5;17:1729375. doi: 10.3389/fpls.2026.1729375 (PMC12916681; doi:10.3389/fpls.2026.1729375)
Supplement: Supplementary file 1 [file DataSheet1.pdf]

Supplementary Materials

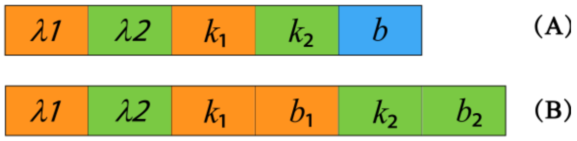

Supplementary Figure S1. Chromosome encoding schemes of spectral indices used in the GA optimization, illustrating the representation of wavelength pairs (λ1, λ2) for (A) the linearly transformed difference spectral index (ltDSI) and (B) the linearly transformed normalized difference spectral index (ltNDSI) and ratio spectral index (ltRSI).

Supplementary Table S2. Calibration performance of SSC prediction models based on GA-optimized linearly transformed reflectance spectral indices (ltDSI, ltNDSI, and ltRSI) across ten independently generated calibration datasets.

Dataset	Spectral index	Wavelength (nm)	R_c^2	RMSEC (°Brix)
1	ltDSI	805, 835	0.836	0.607
	ltNDSI	805, 835	0.832	0.613
	ltRSI	805, 835	0.824	0.628
2	ltDSI	805, 835	0.805	0.630
	ltNDSI	805, 835	0.805	0.631
	ltRSI	805, 845	0.800	0.638
3	ltDSI	805, 835	0.815	0.626
	ltNDSI	805, 835	0.815	0.627
	ltRSI	805, 835	0.814	0.628
4	ltDSI	835, 805	0.825	0.630
	ltNDSI	835, 805	0.823	0.633
	ltRSI	805, 835	0.828	0.624
5	ltDSI	845, 805	0.820	0.617
	ltNDSI	805, 845	0.822	0.614
	ltRSI	805, 845	0.821	0.615
6	ltDSI	835, 805	0.812	0.643
	ltNDSI	805, 835	0.813	0.643
	ltRSI	805, 835	0.813	0.642
7	ltDSI	835, 805	0.853	0.570
	ltNDSI	805, 835	0.852	0.573
	ltRSI	805, 835	0.852	0.573
8	ltDSI	835, 805	0.817	0.622
	ltNDSI	935, 715	0.800	0.650
	ltRSI	845, 805	0.815	0.626
9	ltDSI	835, 805	0.835	0.596
	ltNDSI	805, 845	0.826	0.611
	ltRSI	855, 805	0.823	0.618
10	ltDSI	805, 835	0.831	0.600
	ltNDSI	835, 805	0.830	0.602
	ltRSI	805, 835	0.831	0.601

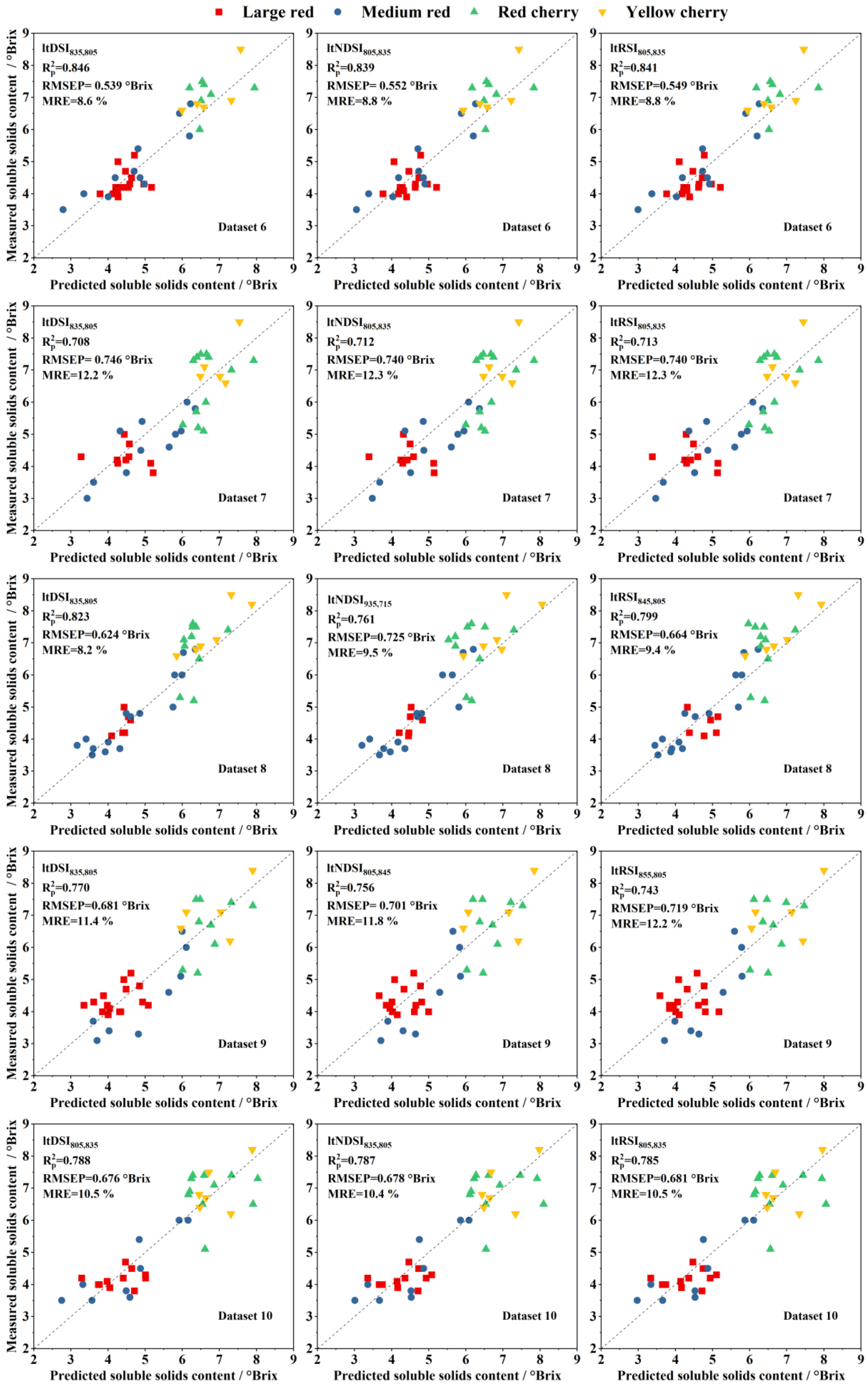

Supplementary Figure S3. Relationships between GA-optimized predicted and measured SSC values for validation Datasets 6–10, with the dashed line indicating the 1:1 reference.

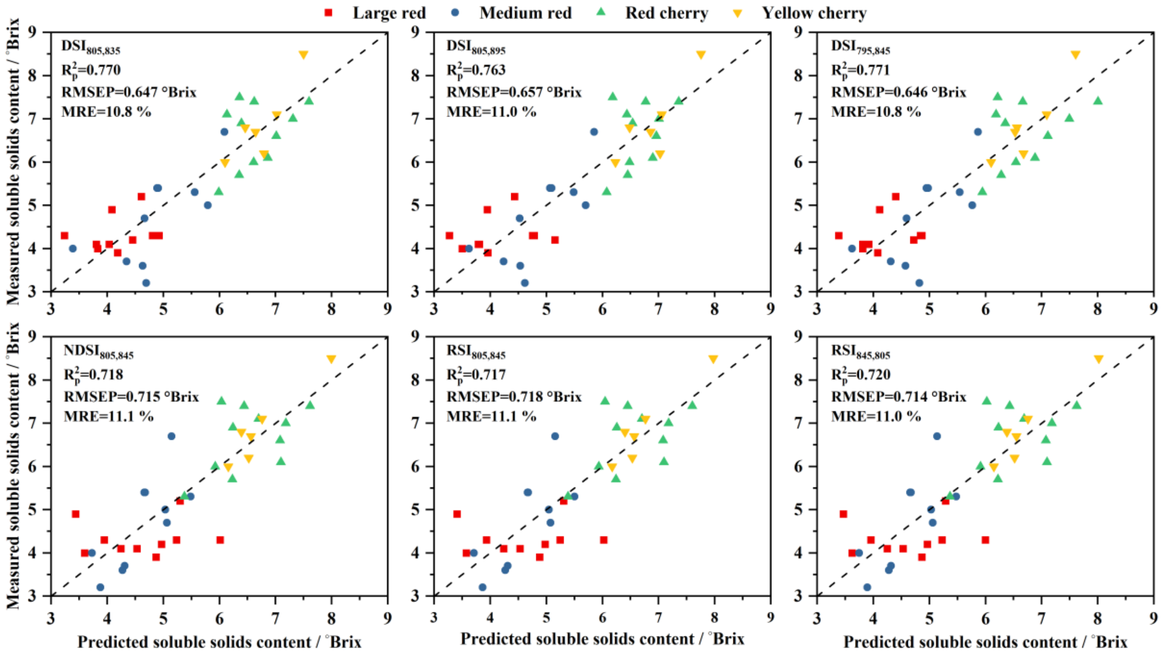

Supplementary Figure S4. Correlations between predicted and measured SSC values obtained using selected conventional spectral index–based models, with the 1:1 reference line shown.

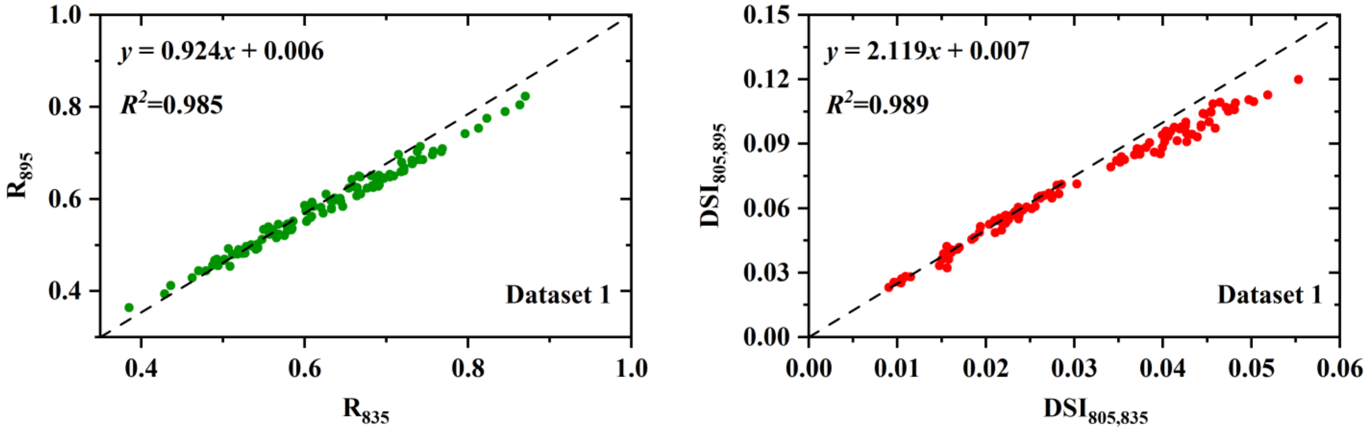

Supplementary Figure S5. Linear relationships between reflectance at 895 nm (R_{895}) and 835 nm (R_{835}), and between the spectral indices $DSI_{805,\ 895}$ and $DSI_{805,\ 835}$.
